# Artificial sweeteners and risk of cardiovascular diseases in the prospective NutriNet-Santé cohort

**DOI:** 10.1093/eurpub/ckac129.013

**Published:** 2022-10-25

**Authors:** C Debras, E Chazelas, L Sellem, C Julia, E Kesse-Guyot, B Allès, M Deschasaux-Tanguy, I Huybrechts, B Srour, M Touvier

**Affiliations:** 1 Nutritional Epidemiology Research Team, Sorbonne Paris Nord University, Bobigny, France; 2 NACRe Network, INRAE, Jouy-en-Josas, France; 3 Public Health Department, Avicenne Hospital, Bobigny, France; 4 International Agency for Research on Cancer, WHO, Lyon, France

## Abstract

**Background:**

Artificial sweeteners are widely used today by the food industry as sugar alternatives. Potential adverse effects of these food additives on cardiovascular disease (CVD) have been suggested in experimental studies, but data from studies involving humans remain very limited. Previous cohorts have focused on artificially sweetened beverages. Our objective was to study the associations between artificial sweeteners from all dietary sources, overall and by molecule (aspartame, acesulfame-potassium and sucralose), and risk of CVDs (overall, coronary heart and cerebrovascular).

**Methods:**

The study included 103,388 participants of the web-based NutriNet-Santé cohort (2009-2021). Artificial sweetener intakes were assessed using repeated 24h dietary records including names and brands of industrial products consumed. Multi-adjusted Cox proportional hazard models were performed. Exposure to artificial sweeteners were coded as 3-category variables: non-consumers, lower consumers (artificial sweetener intake below the sex-specific median) and higher consumers (above the sex-specific median).

**Results:**

Compared to non-consumers, higher consumers of total artificial sweeteners had increased risk for CVD (n = 1502 incident cases, HR = 1.17 [1.01-1.35], P-trend=0.04) and more specifically cerebrovascular diseases (n = 777, HR = 1.34 [1.10-1.62], P = 0.004). Higher consumption of aspartame was associated with increased cerebrovascular diseases (HR = 1.29 [1.03-1.60], P = 0.01). Higher consumption of acesulfame-K was associated with a higher risk of CVD (HR = 1.24 [1.04-1.47], P = 0.02) and cerebrovascular diseases (HR = 1.29 [1.02-1.64], P = 0.1). No association was detected for coronary heart diseases (n = 730 incident cases).

**Conclusions:**

These findings suggest a direct association between higher artificial sweetener consumption and increased CVD risk, in particular cerebrovascular. These results provide key novel information for the ongoing re-evaluation of sweeteners by the European Food Safety Authority.

**Key messages:**

• In this large-scale prospective cohort (n = 103,388), artificial sweeteners (especially aspartame and acesulfame-K) were associated with increased risks of cardiovascular and cerebrovascular diseases.

• These results provide key insights to feed EFSA’s expertise for the ongoing risk assessment of artificial sweeteners.

